# Refining Timely Diagnosis of Early Syphilis by Using Treponema pallidum PCR or IgM Immunoblotting Next to Conventional Serology for Syphilis

**DOI:** 10.1128/jcm.00112-23

**Published:** 2023-05-24

**Authors:** Jacky Flipse, Anne-Marie Niekamp, Anne Dirks, Nicole H. T. M. Dukers-Muijrers, Christian J. P. A. Hoebe, Petra Wolffs, Inge H. M. van Loo

**Affiliations:** a Department of Medical Microbiology, Infectious Diseases, and Infection Prevention, Maastricht University Medical Centre, Care and Public Health Research Institute, Maastricht, the Netherlands; b Department of Sexual Health, Infectious Diseases, and Environmental Health, Living Lab Public Health, Public Health Service South Limburg, Heerlen, the Netherlands; c Department of Social Medicine, Maastricht University, Care and Public Health Research Institute, Maastricht, the Netherlands; d Department of Health Promotion, Maastricht University, Care and Public Health Research Institute, Maastricht, the Netherlands; Marquette University

**Keywords:** *Treponema pallidum*, PCR, serology, public health, STI clinic, syphilis, IgM immunoblot

## Abstract

Treponema pallidum subsp. *pallidum* is a fastidious spirochete and the etiologic agent of syphilis, a sexually transmitted infection (STI). Syphilis diagnoses and disease staging are based on clinical findings and serologic testing. Moreover, according to most international guidelines, PCR analysis of swab samples from genital ulcers is included in the screening algorithm where possible. It has been suggested that PCR might be omitted from the screening algorithm due to low added value. As an alternative to PCR, IgM serology might be used. In this study, we wanted to establish the added value of PCR and IgM serology for diagnosing primary syphilis. Added value was defined as finding more cases of syphilis, preventing overtreatment, or limiting the extent of partner notification to more recent partners. We found that both PCR and IgM immunoblotting could aid the timely diagnosis of early syphilis in ~24% to 27% of patients. PCR has the greatest sensitivity and can be applied to cases with an ulcer with suspected reinfection or primary infection. In the absence of lesions, the IgM immunoblot could be used. However, the IgM immunoblot has better performance in cases with suspected primary infection than in reinfections. The target population, testing algorithm, time pressures, and costs should determine whether either test provides sufficient value to be implemented in clinical practice.

## INTRODUCTION

Treponema pallidum subsp. *pallidum* is a fastidious spirochete and the etiologic agent of syphilis, a sexually transmitted infection (STI). In the Netherlands, the prevalence of syphilis, based on STI clinic data, is 2.9% among men who have sex with men (MSM), 0.1% among women, and 0.5% among heterosexual men (1). Syphilis is almost exclusively (96%) diagnosed among MSM at STI clinics (1).

Syphilis diagnoses and disease staging are based on clinical findings and serologic testing. Moreover, according to most international guidelines, PCR analysis of swab samples from genital ulcers is included in the screening algorithm where possible. It has been suggested that PCR might be omitted from the screening algorithm in Australia due to low added value (2). However, this may lead to missed cases, since up to 30% of cases of primary syphilis present with negative serologic findings (3–11). As an alternative to PCR, IgM serology might be used, because three-fourths of primary syphilis cases have elevated IgM titers (12). Others noted, however, that only one-third of PCR-positive early syphilis cases had positive IgM findings (13). In light of these contrasting findings, we wanted to establish the value of PCR and IgM serology for diagnosing primary syphilis.

The two tests were primarily assessed regarding their added value next to conventional serology. In this study, added value was defined as finding more cases of syphilis or preventing overtreatment (reduced antibiotic regimen [14] or limiting the extent of partner notification to more recent partners [15]). Optimal diagnostic work-up also prevents unnecessary return consultations for confirmatory tests, as not all patients return for follow-up consultations. Thus, our aim was to inform optimal policy by assessing the added value of a positive PCR result and/or IgM immunoblot in the early diagnosis of syphilis.

## MATERIALS AND METHODS

The study population included all 182 STI consultations for 171 unique patients between January 2012 and December 2018 with concurrent T. pallidum PCR and serological testing. These clients of the South Limburg STI clinic (Public Health Service) were tested for the presence of an STI because of symptoms or high-risk sexual behavior (e.g., MSM or sex workers) or because of partner notification. This study was approved and registered by the local ethics committee [local medical ethics committee of the Maastricht University Medical Center] (approval number METC 2018-0579).

Diagnosis and staging of syphilis were performed on the basis of the combination of clinical symptoms, history, serological results, and PCR results. Syphilis was diagnosed in 42% of the STI consultations (77/182 consultations); 64 patients had syphilis stage I, 12 had latent syphilis, and 1 had syphilis of unknown stage.

Clinical evaluation and determination of the added value of PCR and IgM blots were performed by a physician specialized in sexual health. For each consultation, the electronic patient record was reevaluated, and two conclusions were drawn, one based on serologic results and recorded clinical symptoms without PCR results and one with inclusion of the PCR results. The difference between the two evaluations was the added value of the PCR, which was classified as “no difference,” “no consequence,” “missed early (re)infection,” “better staging,” or “avoids retesting.” The IgM blots were tested retrospectively and subsequently related by a clinical microbiologist (physician) to the diagnosis made by the physician specialized in sexual health on the basis of serologic results and clinical symptoms only.

Treponemal serology was performed according to a standard reverse algorithm (16), using a treponemal IgG/IgM electrochemiluminescence immunoassay (ECLIA) for screening (Cobas 8000, product number 07802960; Roche). Positive results were confirmed with Fluorescent treponemal antibody absorption (FTA-abs, Trepo-spot IF, Biomerieux) or IgG immunoblot (product number 5170; Mikrogen), rapid plasma reagin (RPR) reditest (product number 3000-5511; BioKit) and a T. pallidum particle agglutination assay (product number 201633; Fujirebio Inc.), according to the manufacturer's instructions.

IgM immunoblotting (product number 5179; Mikrogen) was performed according to the manufacturer’s instructions. The immunoblot contains 6 pathogen-specific recombinant Treponema antigens (Tp47, Tp17, Tp15, TmpA, Tp257 [Gpd], and Tp453) (for their functions and molecular weights, see Table S1 in the supplemental material). The test is considered positive if ≥2 of these antigens show an intensity equivalent to or stronger than that of the cutoff band.

Tested samples included a subset of the test population (84/183 samples [46%]); 67/84 cases (79%) were (presumptive) primary infections, and 17/84 cases were suspected reinfections. An initial analysis revealed better performance in cases with suspected primary infections, compared with reinfections. Therefore, we oversampled primary infections (i.e., 79% of the included samples versus 69% [126/182 samples] in the original data set). Additionally, a limited panel of sera was included to test for cross-reactivity, consisting of cases with primary Epstein-Barr virus (EBV) (known to cause a nonspecific IgM reaction in the IgM immunoblot, *n* = 3), cytomegalovirus (CMV) (*n* = 3), or Borrelia burgdorferi (*n* = 3) infections, as well as rheumatoid factor-positive samples (*n* = 5), which, according to the manufacturer, should not show any cross-reactivity with the blot.

PCR was performed with swab samples from genital ulcers as described previously (3, 17), detecting the polymerase A gene (*polA*) or the gene for the 47-kDa lipoprotein (*tpp47*). Lesions were probed with dry cotton-tipped swabs. Dry swab samples were sent to the laboratory and vortex-mixed in 600 μL phosphate-buffered saline (PBS). Two hundred microliters was used for DNA purification with the QIAamp DNA minikit (product number 51306; Qiagen, Hilden, Germany) according to the manufacturer’s protocol. Murine CMV glycoprotein B was used as the internal control for extraction and amplification.

The limit of detection was determined using a plasmid containing the PCR amplicon of interest; PCR fragments were ligated into the pGEM-T Easy vector (Invitrogen) and subsequently transformed into Escherichia coli DH10β cells. Transformants were Sanger sequenced to confirm the integrity of the inserts, which resulted in two plasmids harboring an insertion of either 105 bp (*polA*) or 68 bp (*tpp47*). The purified plasmid DNA concentration was measured with a Qubit 3 (Invitrogen) fluorometer, and copy numbers were calculated. For each plasmid, 10 independent dilution series were tested on 9 days, and the limit of detection was determined as the concentration that was detected in each dilution series (i.e., 100% detection).

## RESULTS

### Study population.

The 171 patients included 133 male patients (78%) and 38 female patients (22%). The median age was 30 years (interquartile range [IQR], 22.5 to 44.5 years). Most patients were Dutch (144/171 patients [83%]). Sexual preferences were reported as heterosexual (71/171 patients [42%]), MSM (88/171 patients [52%]), or bisexual (12/171 patients [7%]). The 182 consultations concerned 175 ulcers, 6 condylomata, and 1 undefined lesion. Fifty-six consultations concerned cases with a history of syphilis, and 126 cases did not have a history of syphilis. The electronic patient records did not contain information regarding the duration of symptoms.

### Validation of the PCRs.

First, we clinically validated two PCRs for detection of T. pallidum in swab samples from ulcers; one PCR targeted the *polA* gene (17), and the other PCR targeted the *tpp47* gene (3). A head-to-head comparison showed 98% agreement (94/96 samples) and 98% sensitivity (43/44 samples) between the two PCRs, and each PCR had 99% agreement with the external reference laboratory (GGD Amsterdam, Amsterdam, the Netherlands), which employs the same *polA*-directed PCR. In our hands, the smallest amount that could be detected with 100% probability was 20 to 42 copies per reaction (i.e., 360 to 756 copies per cotton tip). Thereafter, routine diagnostic analysis was performed using the *polA* PCR, while the *tpp47* PCR served as the back-up for suspected false-negative or false-positive results, at the discretion of the laboratory specialist. In our laboratories, this specialist is a medical molecular microbiologist, a registered specialist in the Netherlands. Importantly, the two PCRs showed similar sensitivity and specificity, irrespective of the target gene, and the threshold cycle (*C_T_*) values did not differ significantly between the two PCRs (Δ*C_T_* for the *tpp47* PCR versus the *polA* PCR, −0.21 [*n* = 37]).

### Clinical evaluation of PCR.

We assessed the clinical value of the *polA* PCR in each of the 182 consultations (Table 1). Sixty-six of the PCRs (66/182 PCRs [36%]) were positive for T. pallidum (64 ulcers/lesions and 2 condylomata). Seven of the positive PCRs (7/66 PCRs [11%]) detected cases of early syphilis missed by standard diagnostics. Nine of the positive PCRs (9/66 PCRs [14%]) aided in the staging of syphilis, thus reducing the duration of treatment and the extent of retrospective partner notification. Twenty-seven of the negative PCRs (27/116 PCRs [23%]) nullified the need to recall the patient for confirmatory testing. Overall, 24% (43/183 consultations) benefitted from having a T. pallidum PCR next to serology. In other cases, the consulting clinician already strongly suspected syphilis based on the clinical symptoms (e.g., exanthema) and other personal risk factors pertaining to the case (e.g., the case was notified by a syphilis-positive sex partner).

**TABLE 1:** Generalized overview of standard work-up in the STI clinic based on syphilis serology and PCR^*a*^

Symptom(s)	Ulcers/condylomata?	History of syphilis?	Serologic result^*b*^	PCR result	No. of cases					
					Total	No difference	No consequence	Missed early (re)infection	Better staging	Avoids retesting
Yes	Yes	Yes	ΔRPR ≥4-fold	Positive	22	13	4	0	5	0
				Negative	2	2	0	0	0	0
			ΔRPR <4-fold	Positive	4	1	0	3	0	0
				Negative	28	16	1	0	0	11
		No	Positive	Positive	36	20	12	0	4	0
				Negative	3	0	3	0	0	0
			Inconclusive	Positive	0	0	0	0	0	0
				Negative	3	0	0	0	0	3
			Negative	Positive	4	0	0	4	0	0
				Negative	81	68	0	0	0	13
	No	No PCR done; diagnosis based on serology and history								
No	No PCR done; diagnosis based on serology and history									

a In general, the diagnosis and staging of syphilis depended on several parameters, i.e., (i) whether the patient has symptoms, (ii) whether lesions are present or absent, and (iii) whether the patient has a history of syphilis. The added value of PCR was evaluated for each case. In cases in which the PCR result did not contribute to diagnosis or treatment, the effect was “no difference.” In cases in which a specific diagnosis was already strongly suspected and the patient was treated accordingly irrespective of results, the effect was “no consequence.” If syphilis was missed without PCR, then the case was flagged as “missed early (re)infection.” “Better staging” indicates that PCR led to better staging and thus reduced duration of treatments and/or reduced retrospective partner notification. “Avoids confirmatory testing” indicates that the addition of PCR results to serologic results reduced the need for recalling patients for follow-up serology prior to definite diagnosis.

b ΔRPR ≥4-fold indicates an increase in the RPR titer of ≥2 dilution steps; ΔRPR <4-fold indicates that the RPR titer did not increase ≥4-fold.

Of the 70 patients with serologic results indicative of syphilis stage I (i.e., seroconversion or ≥4-fold increase [2 dilutions] in RPR titer), 8 cases (8/70 cases [11%]) were PCR negative. These cases were diagnosed with late latent syphilis (*n* = 1), recent latent syphilis (*n* = 3), lues stage I (*n* = 3), syphilis of unknown stage, or endemic treponematosis (*n* = 1).

The characteristics of the PCR relative to the combination of clinical assessment and serology are as follows: sensitivity, 89% (65/73 cases); specificity, 99% (108/109 cases); positive predictive value (PPV), 99% (65/66 cases); negative predictive value (NPV), 93% (108/116 cases). The test characteristics were not significantly different between syphilis-naive cases and cases with a history of syphilis (Table 2).

**TABLE 2:** Correlation between PCR results and clinical diagnosis of syphilis

Patient group and PCR results	No. of cases with clinical diagnosis of syphilis^*a*^		
	Positive	Inconclusive^*b*^	Negative
All cases			
Positive	59	6	1
Negative	8	21	87
History of syphilis			
Positive	36	3	1
Negative	4	12	70
No history of syphilis			
Positive	23	3	0
Negative	4	9	17

a Clinical diagnosis is the combination of clinical assessment and serology.

b Inconclusive diagnosis can indicate either inconclusive serology (*n* = 2) or inconclusive clinical assessment. The characteristics of the PCR relative to diagnosis based on serology and clinical assessment alone were as follows: sensitivity, 89.0% (65/73 cases); specificity, 99% (108/109 cases); PPV, 99% (65/66 cases); NPV, 93% (108/116 cases). When the results are separated into suspected reinfections (i.e., history of syphilis) versus suspected primary infections, the characteristics are as follows: for reinfections, sensitivity, 91% (39/43 cases); specificity, 99% (82/83 cases); PPV, 98% (39/40 cases); NPV, 95% (82/86 cases); for suspected primary infections, sensitivity, 87% (26/30 cases); specificity, 100% (26/26 cases); PPV, 100% (26/26 cases); NPV, 87% (26/30 cases).

### IgM immunoblotting.

Because not all cases with suspected syphilis present with an ulcer, PCR cannot always be performed. Thus, we assessed whether an IgM immunoblot could supplement conventional serology to aid the diagnosis.

A subset of the test population (84/182 consultations [46%]) described above was additionally tested with an IgM immunoblot. We determined that both positive and indeterminate IgM blots should be considered positive, as this yielded highest sensitivity; 6/8 patients (75%) with indeterminate IgM blots had a clinical diagnosis of syphilis. Here, indeterminate blots are referred to as positive. One patient had a positive treponemal IgM/IgG screening result without confirmation with the nontreponemal tests, IgM immunoblot, or PCR. There was no follow-up consultation; therefore, the diagnosis remained uncertain. This patient was excluded from further analyses.

Initial analysis revealed that 2 of 14 samples tested for cross-reactivity showed a positive IgM immunoblot, i.e. 1 positive for EBV and 1 positive for CMV, with both being heterophile antibody positive. This is known to give nonspecific IgM reactivity in the IgM immunoblot.

Table 3 depicts the results of the IgM immunoblot in cases with either primary syphilis or reinfection, based on clinical assessment, IgG serology, and PCR. The characteristics of the IgM blot relative to the diagnosis are as follows: sensitivity, 80% (40/50 cases); specificity, 94% (31/33 cases); PPV, 95% (40/42 cases); NPV, 76% (31/41 cases). When the results are separated into those for suspected primary infections versus reinfections, the characteristics are as follows: for suspected primary infections, sensitivity, 91% (30/33 cases); specificity, 94% (30/32 cases); PPV, 94% (30/32 cases); NPV, 91% (30/33 cases); for reinfections, sensitivity, 59% (10/17 cases); specificity, 100% (1/1 case); PPV, 100% (10/10 cases); NPV, 13% (1/8 cases).

**TABLE 3:** Results of IgM immunoblotting in the population of the STI clinic in South Limburg, the Netherlands, with clinical symptoms of ulcers/condylomata

History of syphilis?	Serologic result^*a*^	Active syphilis?^*b*^	No. with IgM immunoblot result of:	
			Positive	Negative
Yes	ΔRPR ≥4-fold	Yes	8	3
		No	0	0
	ΔRPR <4-fold	Yes	2	4
		No	0	1
No	Positive	Yes	26	2
		No	0	0
	Negative	Yes	4	1
		No	2	30

a ΔRPR ≥4-fold indicates an increase in the RPR titer of ≥2 dilution steps. ΔRPR <4-fold indicates that the RPR titer did not increase ≥4-fold.

b Diagnosis of syphilis is based on a combination of serologic, clinical assessment, and PCR results.

Next, we compared the performance of the IgM immunoblot to the PCR for patients (see Table S2 in the supplemental material) and evaluated the IgM immunoblot and PCR against the final diagnosis based on clinical assessment and conventional serology (Table 4). The T. pallidum PCR and IgM immunoblot showed substantial agreement (Cohen’s kappa, 0.85) (see Table S2).

**TABLE 4:** Comparison of IgM immunoblotting and PCR in relation to the clinical diagnosis based on serology and clinical assessment only for patients with clinical symptoms of ulcers/condylomata

History of syphilis?	Serologic result^*a*^	Active syphilis?^*b*^	Proportion (%) positive (no. positive/total no.)	
			IgM immunoblot	PCR
Yes	ΔRPR ≥4-fold	Yes	73 (8/11)	82 (9/11)
		No	0	0
	ΔRPR <4-fold	Yes	50 (1/2)	50 (1/2)
		Inconclusive	20 (1/5)	60 (3/5)
		No	0	0
No	Positive	Yes	93 (26/28)	93 (26/28)
		No	0	0
	Negative	Yes	100 (1/1)	0 (0/1)
		Inconclusive	50 (3/6)	50 (3/6)
		No	7 (2/30)	3 (1/30)

a ΔRPR ≥4-fold indicates an increase in the RPR titer of ≥2 dilution steps. ΔRPR <4-fold indicates that the RPR titer did not increase ≥4-fold.

b Diagnosis of syphilis is based on a combination of serologic and clinical assessment results.

As can be seen in Table 4, 4 (36%) of 11 patients with an uncertain (inconclusive) syphilis diagnosis had positive IgM immunoblot results. One patient did not have syphilis based on PCR and/or follow-up serologic results. Thus, the IgM blot added to the diagnosis for 3/11 patients (27%), while it overdiagnosed 1 patient (9%).

## DISCUSSION

In this study, the added value of PCR and IgM immunoblotting for the timely diagnosis of early syphilis, next to conventional serology, was investigated in 182 STI clinic consultations (171 unique patients).

Two PCRs, targeting the *polA* and *tpp47* genes, were selected and validated to be used in routine diagnostics. The two PCRs showed similar sensitivity, irrespective of the PCR target, which is in line with previous reports (18, 19); however, a recent article showed better sensitivity for the *polA* PCR than the *tpp47* PCR (11). When conventional serology was combined with a T. pallidum PCR, the PCR added value to 24% of the consultations concerning cases with a suspected syphilitic ulcer; the PCR detected 7 additional syphilis cases (+11%) and either nullified the need to recall patients to the STI clinic for confirmatory testing (14%) or reduced the duration of treatment and the extent of retrospective partner notification (5%).

The benefit of including a T. pallidum PCR likely depends on whether patients present for diagnosis within the window period of the serologic response. Brischetto et al. evaluated their workflow in the Australian setting with on-site serology and PCR in a central laboratory at a distant site. The authors concluded that adding PCR to their workflow detected an additional 3% of syphilis cases and had little additional value (2) but considerable monetary and time costs.

Cuba, on the other hand, provides free health care services, and many patients presented within 15 days after formation of an ulcer (11), which is within the window period of serology (20). Consequently, the authors noted that the syphilis PCR significantly contributed to detection of syphilis in the Cuban population, a population that resides where both syphilitic and nonsyphilitic treponemes are endemic (21). In contrast, the nonsyphilitic treponemes are not endemic in the Netherlands; however, here too we observed that the T. pallidum PCR significantly contributed to early detection of syphilis, likely due to the early stage of diagnosis. For example, 35% of our PCR-positive cases had a low RPR titer of ≤4 (see Fig. S1 in the supplemental material), whereas a recent study in Australia found that only 15% of cases had an RPR titer of ≤4 (2).

In the absence of an ulcer on a patient, IgM immunoblotting could be used to supplement the conventional serology. However, our data show that IgM immunoblotting is best used in cases with primary syphilis, where its sensitivity and NPV are high (i.e., 91% [30/33 cases] and 94% [30/32], respectively). In that scenario, the IgM immunoblot results were positive for the majority of patients (93%), which is even higher than the 79% described previously (12).

The poor sensitivity of IgM serology among patients with syphilis reinfection was shown previously (12, 13). Moreover, there is a risk of false-positive results with primary EBV and CMV (heterophilic antibody positive). Because the clinical symptoms of the diseases are quite different, however, we do not expect this cross-reactivity to result in clinical dilemmas. Therefore, the test is best used in cases of suspected primary infection in which serology and clinical assessment lead to an inconclusive diagnosis of syphilis. This requires close collaboration between the clinician and the laboratory, which is not considered feasible in routine diagnostics. Thus, in our study population, the inclusion of PCR would be the primary choice. Furthermore, the relationship between IgM antibodies and disease activity, disease stage, and status after treatment is under investigation. Currently, the only use is in congenital syphilis (22, 23).

Noda and colleagues provided a good overview of all of the pitfalls in the laboratory diagnosis of syphilis (11). We highlight some pitfalls and solutions regarding this study. Risks regarding false-positive and false-negative results produced during the diagnostic processes are checked by the use of internal and external controls. Laboratory contamination is a possibility if high-positive and low-positive samples are included in the same test, prompting retesting of the low-positive samples for confirmation.

False-negative results due to inaccurate swabbing not only reflect the actual technique but also may occur if the ulcer has almost healed. Lastly, clinically false-positive results could result from infection with nonsyphilitic T. pallidum subspecies (i.e., T. pallidum subsp. *endemicum*, T. pallidum subsp. *pertenue*, and T. pallidum subsp. *carateum*). Cross-reactivity between the T. pallidum subspecies cannot be avoided, since neither PCR nor serology can distinguish between syphilitic and nonsyphilitic treponematoses (11, 24). While these T. pallidum subspecies are not endemic to the Netherlands (24), cross-reactivity should be considered in cases with a history in areas in which the subspecies are endemic.

In short, both PCR and IgM immunoblotting can facilitate the diagnosis of early syphilis for approximately ~24% to 27% of patients. PCR has the greatest sensitivity and can be applied to cases of suspected reinfection or primary infection; however, it depends on the presence of an ulcer. IgM immunoblotting has better performance in cases of suspected primary infection (91%) than in cases of reinfection (59%) and does not rely on the presence of an ulcer. However, false-positive results are a concern. The target population, testing algorithm, time pressures, and costs should determine whether either test provides sufficient value to be implemented in clinical practice.
