# Supplementary material for: Refining Timely Diagnosis of Early Syphilis by Using Treponema pallidum PCR or IgM Immunoblotting Next to Conventional Serology for Syphilis
Source: J Clin Microbiol. 2023 May 24;61(6):e00112-23. doi: 10.1128/jcm.00112-23 (PMC10281170; doi:10.1128/jcm.00112-23)
Supplement: Supplemental file 1 — Supplemental material. Download jcm.00112-23-s0001.docx, DOCX file, 0.06 MB [file jcm.00112-23-s0001.docx]

**Supplemental information for**

Refining timely diagnosis of early syphilis by using *Treponema pallidum* PCR or IgM immunoblot next to conventional serology for syphilis.

Jacky Flipse^a,b * #^, Anne-Marie Niekamp^c,d *^, Anne Dirks^a^, Nicole H.T.M. Dukers-Muijrers^c,e^, Christian J.P.A. Hoebe^a,c,d^, Petra Wolffs^a^, Inge H.M. van Loo^a #^

^a.^ Department of Medical Microbiology, Infectious Diseases & Infection Prevention, Maastricht University Medical Centre (MUMC+), Care and Public Health Research Institute (CAPHRI), Maastricht, the Netherlands.

^b.^ present address: Laboratory for Medical Microbiology and Immunology, Rijnstate, Velp, the Netherlands

^c.^ Department of Sexual Health, Infectious Diseases and Environmental Health, Living Lab Public Health, Public Health Service South Limburg, Heerlen, the Netherlands

^d.^ Department of Social Medicine, Maastricht University, Care and Public Health Research Institute (CAPHRI), Maastricht, the Netherlands.

^e.^ Department of Health Promotion, Maastricht University, Care and Public Health Research Institute (CAPHRI), Maastricht, the Netherlands.

# correspondence to: Jacky Flipse (jflipse@rijnstate.nl); Inge van Loo (ihm.van.loo@mumc.nl)

* shared first authors

Table S1. Recombinant antigens in the IgM blot, their function and molecular weight.

| Recombinant antigen | Function | Size (kDa) |
| --- | --- | --- |
| Tp 47 | Membrane protein | 47 |
| TmpA | Membrane protein | 42 |
| Tp257 (Gpd) | Glycerophosphodiester-Phosphodiesterase, Membrane protein | 39 |
| Tp453 | Membrane protein | 28 |
| Tp 17 | Membrane protein | 17 |
| Tp 15 | Membrane protein | 15 |

Source: Package Insert *recom*Line Treponema IgM, article number 5179, version GARLTP003EN_2013-04. See also : https://www.mikrogen.de/english/products/product-overview/testsystem/treponema-igg-1.html (date accessed: April 9^th^, 2023).

Table S2. Comparison of IgM immunoblot and PCR.

|  | | IgM immunoblot | |
| --- | --- | --- | --- |
|  |  | Positive | Negative |
| PCR | Positive | 36 | 7 |
|  | Negative | 6 | 35 |


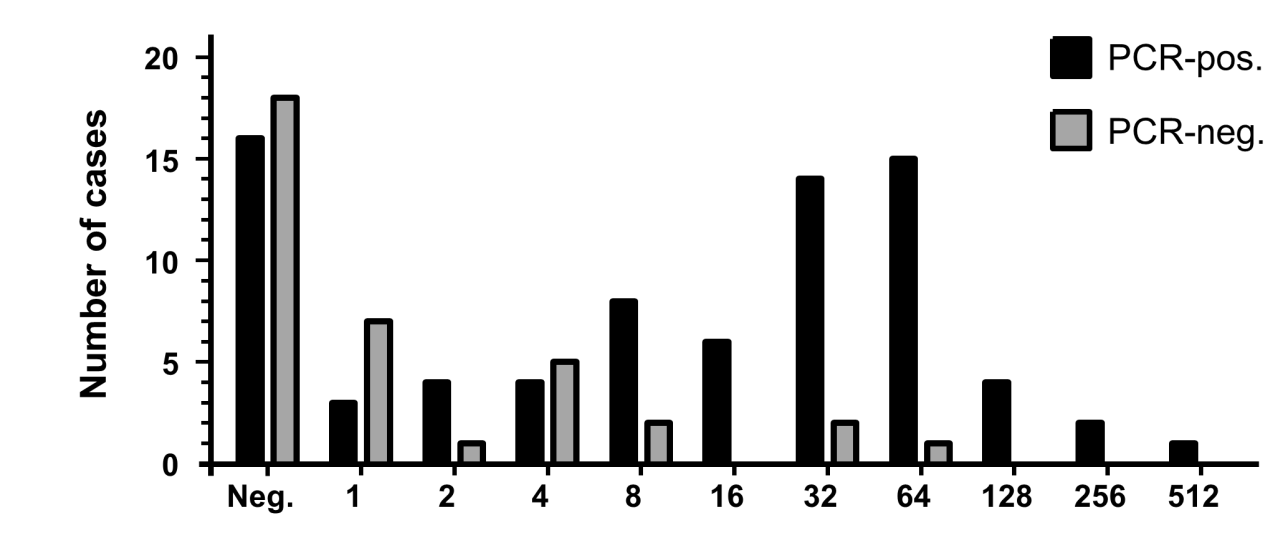


Figure S1: Distribution of RPR values among the Syphilis serology positive cases splitted in PCR-positive and PCR-negative cases. Ten out of twenty-nine (35%) PCR-positive cases had an RPR of ≤4. RPR: Rapid Plasma Reagin. Neg.: negative/non-reactive.
